# Resveratrol Inhibits MMP3 and MMP9 Expression and Secretion by Suppressing TLR4/NF-*κ*B/STAT3 Activation in Ox-LDL-Treated HUVECs

**DOI:** 10.1155/2019/9013169

**Published:** 2019-09-10

**Authors:** Ming Zhang, Yun Xue, Huilian Chen, Lingbing Meng, Beidong Chen, Huan Gong, Yanyang Zhao, Ruomei Qi

**Affiliations:** ^1^MOH Key Laboratory of Geriatrics, Beijing Hospital, National Center of Gerontology, Beijing, China; ^2^Graduate School of Peking Union Medical College, Beijing, China

## Abstract

**Aim:**

Resveratrol is a natural plant polyphenol. The present study investigated the effects of resveratrol on the Toll-like receptor 4- (TLR4-) mediated expression and secretion of matrix metalloproteinases (MMPs) in oxidized low-density lipoprotein- (ox-LDL-) treated human umbilical vein endothelial cells (HUVECs).

**Methods:**

Protein expression was analyzed by immunoblotting. The secretion of MMPs was measured by an enzyme-linked immunosorbent assay. The animal experiments were performed with and without resveratrol treatment in high-fat chow-fed mice.

**Results:**

Resveratrol inhibited the expression of TLR4, MMP3, and MMP9 in ox-LDL- and lipopolysaccharide- (LPS-) treated HUVECs. Resveratrol reduced the secretion of MMP3 and MMP9 that was induced by ox-LDL and LPS. The TLR4 inhibitor CLI-095 similarly suppressed the expression and secretion of MMP3 and MMP9 in ox-LDL- and LPS-treated HUVECs. Resveratrol attenuated the phosphorylation of the transcription factors nuclear factor-*κ*B (NF-*κ*B) and signal transducer and activator of transcription 3 (STAT3) that was induced by ox-LDL and LPS. Resveratrol recovered Sirt1 expression. In the animal experiments, resveratrol decreased TLR4 expression in the aorta, MMP9 levels in plasma, and vascular structural changes in high-fat chow-fed mice, with no significant effect on plasma MMP3 levels.

**Conclusion:**

Resveratrol inhibited the TLR4-mediated expression and secretion of MMP3 and MMP9 in ox-LDL-treated HUVECs. The mechanism of action of resveratrol may be associated with the suppression of NF-*κ*B and STAT3 phosphorylation and restoration of Sirt1 expression. Resveratrol exerts protective effects against vascular structural changes in high-fat chow-fed mice.

## 1. Introduction

Atherosclerosis is a chronic vascular inflammatory disease that is related to aging and various pathological conditions, such as metabolic disorder and oxidative stress [1–3]. Phenotypic changes in vascular cells in atherosclerosis mainly involve inflammation, but the mechanisms are still unclear. Treatment and prevention strategies are still limited. Oxidized low-density lipoprotein (ox-LDL) is a risk factor for the development of atherosclerosis. Ox-LDL can induce a vascular inflammatory response through endothelial cell damage, promoting the formation of foam cells, proliferation of smooth muscle cells, and stimulation of platelet activation [4–6]. Cells then produce and release inflammatory proteins, chemokines, and cytokines to further promote vascular inflammation in atherosclerosis [7, 8].

Toll-like receptors (TLRs) are innate immune pattern recognition receptors [9, 10]. The primary function of TLRs is the recognition of specific and distinct conserved endogenous and exogenous molecular patterns (danger-associated molecular pattern (DAMP) and pathogen-associated molecular pattern (PAMP)) [11, 12]. Several studies have shown that TLR4 is involved in inflammatory responses in atherosclerosis. TLR4 expression is high in atherosclerosis patients. TLR4 activation is associated with increases in tumor necrosis factor *α* (TNF*α*) and interleukin-1*β* (IL-1*β*) levels [13]. Our previous study found that high glucose enhanced the expression of TLR4, MyD88, and platelet-activating factor receptors in human umbilical vein endothelial cells (HUVECs), and the mechanism involved the JAK2/signal transducer and activator of transcription 3 (STAT3) pathway and mitogen-activated protein kinase phosphorylation [14]. Remaining unknown, however, are TLR4-mediated inflammatory signaling in atherosclerosis and the potential functions.

Matrix metalloproteinases (MMPs) are extracellular zinc proteases that play an important role in the development and progression of atherosclerosis [15–17]. Human MMPs comprise 28 members that provoke focal destruction of the vascular extracellular matrix (ECM) through proteolysis [18]. The components of the ECM include elastin, fibronectin, laminin, and various types of collagens. Recent studies showed that vascular cell signaling in the ECM also plays an important role in the inflammatory response. Matrix metalloproteinase activation is involved in inflammatory cell infiltration, vascular smooth muscle cell (VSMC) migration, ECM remodeling, and neovascularization [19, 20].

Endogenous tissue inhibitor of metalloproteinases (TIMP) has been reported to play a complex dual role during the late-stage progression and rupture of atherosclerotic plaques [21]. However, whether MMP activation relies on TLR4 signaling is less understood.

Resveratrol (3,5,4′-trihydroxystilbene) is a natural plant polyphenol that has been isolated from grapes, peanuts, and berries. Resveratrol has various benefits, such as antioxidative, anti-inflammatory, and antiapoptotic effects, in addition to inhibiting platelet activation and preventing or slowing the progression of several illnesses, including cardiovascular disease and cancer [22, 23]. Resveratrol has been shown to increase Sirt1/5′-adenosine monophosphate kinase (AMPK) activation in endothelial cells and in mice. Sirt1/AMPK signaling is related to the lifespan in both mice and humans. Sirt1 is a protective molecule in cellular and organ function through the activation of peroxisome proliferator-activated receptor-*γ* coactivator 1*α* (PGC-1*α*). Sirt1 also attenuates oxidative stress in metabolic and cardiovascular diseases [24].

The present study investigated the effects of resveratrol on the TLR4-mediated expression and secretion of MMPs in ox-LDL-treated HUVECs and the potential mechanisms. We also evaluated the effects of resveratrol on vascular inflammation in high-fat chow-fed mice.

## 2. Materials and Methods

### 2.1. Ethics Statement

According to the Declaration of Helsinki, umbilical cords were donated by cesarean section patients, from whom we received written informed consent. The study was approved by the Ethics Committee of the Beijing Institute of Geriatrics (no. 201508).

### 2.2. Materials

Resveratrol (>99% purity, determined by high-performance liquid chromatography) was purchased from Sigma-Aldrich (St. Louis, MO, USA). CLI-095 was purchased from Invitrogen (San Diego, CA, USA). Monoclonal anti-MMP3 antibody (#14351), monoclonal anti-MMP9 antibody (#13667), monoclonal anti-phosphorylated NF-*κ*B p65 antibody (#3033), monoclonal anti-NF-*κ*B p65 antibody (#8242), and polyclonal anti-Sirt1 antibody (#8469) were purchased from Cell Signaling Technology (Danvers, MA, USA). Monoclonal anti-TLR4 antibody (sc-293072), monoclonal anti-phosphorylated STAT3 antibody (sc-7993), and polyclonal anti-STAT3 antibody (sc-482) were purchased from Santa Cruz Biotechnology (Santa Cruz, CA, USA). The working concentration of antibodies was 1 : 1000 in Western blotting.

### 2.3. Preparation and Culture of HUVECs

Primary HUVECs were collected from human umbilical veins by digestion in 0.1% collagenase I in M199 medium (Gibco, NY, USA) for 15 min. The solution was collected and centrifuged at 1000 rotations per minute (rpm) for 10 min. The cells were cultured in M199 medium that contained 10% fetal bovine serum (Gibco, NY, USA), 2 mM glutamine, 100 U/ml penicillin, 100 *μ*g/ml streptomycin, and 20 ng/ml endothelial growth factor (R&D, Minneapolis, MN, USA) in an incubator at 37°C and 5% CO_2_. HUVECs from passage 3 were used in the present study.

### 2.4. Cell Viability Assay

Cell Counting Kit-8 was used to measure cell viability. HUVECs were grown in 96-well plates and incubated with various concentrations of resveratrol for 2 hours, then added 10 *μ*l CCK-8 solution at 37°C for 2 hours. Optical density (OD) was measured at 450 nm with a microplate reader (Thermo Scientific, Multiskan FC, USA).

### 2.5. Immunoblotting

HUVECs were incubated with various concentrations of resveratrol (1, 10, and 100 *μ*M) for 1 h and then exposed to ox-LDL (0.1 mg/ml) for 8 h. Cell lysis was performed in lysis buffer (1% Triton X-100, 100 mM Tris/HCl (pH 7.2), 50 mM NaCl, 5 mM ethylenediaminetetraacetic acid (EDTA), 5 mM ethylene glycol tetraacetic acid (EGTA), 1 *μ*M phenylmethylsulfonyl fluoride (PMSF), and 100 *μ*g/ml leupeptin), and lysed cells were then centrifuged at 15000 × *g* at 4°C for 5 min. Protein samples were boiled in sodium dodecyl sulfate (SDS) loading buffer for 5 min, run on SDS-polyacrylamide gel electrophoresis (PAGE), and transferred to a polyvinylidene difluoride (PVDF) membrane. Primary antibody incubations were performed overnight at 4°C. A horseradish peroxidase-conjugated secondary antibody was applied for 1 h at room temperature and developed using a Super Signal developing reagent (Pierce, Thermo Scientific, Rockford, IL, USA). Blot densitometry was then performed, and the bands were analyzed using the Gene Genius Bio Imaging System.

### 2.6. Enzyme-Linked Immunosorbent Assay

To detect the secretion of MMP3, MMP9, and IL-6 in the supernatant, cells were plated in 24-well plates. The cells were then treated with and without resveratrol for 1 h, and then, ox-LDL (0.1 mg/ml) was added for 8 h. After centrifugation at 1000 rpm for 10 min, the supernatant was collected to measure MMP3, MMP9, and IL-6. The levels of secreted MMP3 and MMP9 were determined by an enzyme-linked immunosorbent assay (ELISA) kit (Abcam, USA) according to the manufacturer's instructions. The level of secreted IL-6 was determined by an ELISA kit (Elabscience, Wuhan, China) according to the manufacturer's instructions.

### 2.7. Immunofluorescence

HUVECs were cultured on six-well culture plates. The cells were pretreated with resveratrol (100 *μ*M) for 1 h and then with ox-LDL (0.1 mg/ml) for 8 h. The cells were fixed with 4% paraformaldehyde for 15 min and washed twice. The cells were then incubated with an antibody against TLR4 or NF-*κ*B or STAT3 for 1.5 h. After washing three times, a DyLight-conjugated antibody was added to the cells for 1.5 h at room temperature. The nucleus was stained with DAPI for 5 min at room temperature, and staining was observed under a confocal microscope (Nikon ECLIPSE T*i*) (Nikon, Tokyo, Japan).

### 2.8. Animal Experiments

Eight-week-old and 36-week-old male C57BL/6 mice were obtained from the Experimental Animal Laboratory of Beijing University (Beijing, China). The mice were fed high-fat chow that contained 20% fat and 1.25% cholesterol. The mice were randomly divided into three groups (*n* = 12/group). The control group was given normal chow. The high-fat chow-fed mice were given vehicle (0.3 ml phosphate-buffered saline/day) or resveratrol (22.4 mg/kg/day) intragastrically for 8 weeks [25]. The mice were housed in a room with a 12 h/12 h light/dark cycle and allowed free access to food and water. At the end of the experiments, the animals were sacrificed, and blood samples were collected and analyzed.

### 2.9. Immunohistochemistry

The mice were euthanized with 1.5% isoflurane, and the aorta was dissected. The aortic root specimens were dissected under a microscope, fixed in a 4% formaldehyde solution, and frozen in an optimal-cutting-temperature embedding medium for serial cryosectioning that covered 0.8 mm of the root. The cryosections (6 *μ*m) were examined by immunohistochemistry. The average immunohistochemical staining with specific antibodies was assessed from multiple samples of each artery. A bound antibody was detected with a DAB substrate kit (Beijing Zhongshan Golden Bridge Biotechnology, Beijing, China). Semiquantitative immunostaining analysis was performed using an Olympus microscope that was linked to the Image-Pro Plus image analysis system (Media Cybernetics, Bethesda, MD, USA).

### 2.10. Sirius Red Staining

To observe collagen components, we used Sirius red staining. The Sirius staining kit was purchased from Solarbio (Solarbio Science and Technology Co., Beijing, China). Sections were stained with Weigert iron Sumu essence dye for 15 min, rinsed for 5 min, and then washed with distilled water. The sections were covered with 200 *μ*l Sirius red dye for 1 h. The scanned slides were viewed at 200x magnification with an Olympus microscope with the Nikon CCD system (Tokyo, Japan).

### 2.11. Statistical Analysis

Quantitative data are presented as mean ± SEM. Significant differences between two groups were analyzed using two-tail unpaired Student's *t*-test. All of the analyses were performed using SPSS 18.0 software (Armonk, NY, USA). Values of *p* < 0.05 were considered statistically significant.

## 3. Results

### 3.1. Resveratrol Inhibited TLR4 Expression in Ox-LDL- and LPS-Treated HUVECs

**Figure 1:**
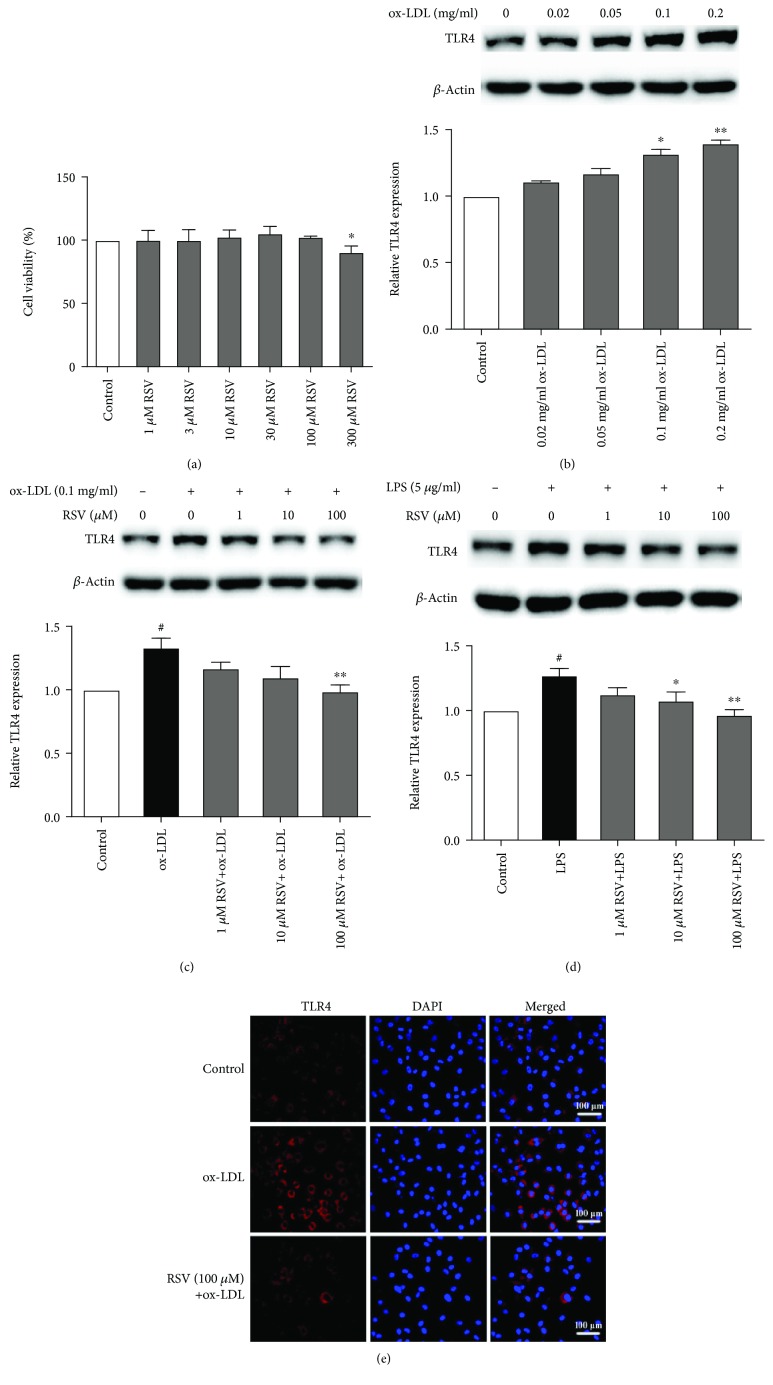
Resveratrol inhibits TLR4 expression in ox-LDL- and LPS-treated HUVECs. The cells were treated with various concentrations of resveratrol for 1 h, and ox-LDL (0.1 mg/ml) or LPS (5 *μ*g/ml) was then added for 8 h. TLR4 expression was analyzed by Western blot and immunofluorescence. The column chart represents the density analysis from three independent experiments. (a) Analysis of cell viability by various concentrations of resveratrol. (b) TLR4 expression that was induced by various concentrations of ox-LDL. (c) Resveratrol inhibited TLR4 expression that was induced by ox-LDL. (d) Resveratrol inhibited TLR4 expression that was induced by LPS. (e) TLR4 expression was determined by immunofluorescence. Resveratrol (100 *μ*M) reduced TLR4 expression in ox-LDL-treated HUVECs. ^#^*p* < 0.05, significant difference between non-ox-LDL-treated cells and ox-LDL-treated cells; ^∗^*p* < 0.05, ^∗∗^*p* < 0.01, significant difference between non-resveratrol-treated cells and resveratrol-treated cells.

TLR4 has been shown to be involved in the inflammatory response in atherosclerosis. To determine whether the protective action of resveratrol involves TLR4 signaling in ox-LDL-treated HUVECs, we first evaluated the effect of resveratrol on TLR4 expression. As shown in Figures 1(a) and 1(b), ox-LDL (0, 0.02, 0.05, 0.1, and 0.2 mg/ml) dose-dependently induced TLR4 expression in HUVECs. Based on this finding, 0.1 mg/ml ox-LDL was used in the subsequent experiments. Ox-LDL (0.1 mg/ml) increased TLR4 expression by 33.2%, and resveratrol (100 *μ*M) significantly abolished ox-LDL-induced TLR4 expression. Next, we used LPS (a specific TLR4 agonist) to evaluate the effect of resveratrol on TLR4 in HUVECs. As shown in Figure 1(c), LPS treatment increased TLR4 expression by 27.1%, and resveratrol dose-dependently attenuated phenotypic changes. We used immunofluorescence to observe TLR4 expression. The membrane expression of TLR4 increased in ox-LDL-treated HUVECs compared with controls, and resveratrol treatment suppressed this effect that was induced by ox-LDL (Figure 1(d)).

### 3.2. Resveratrol Inhibited MMP3 and MMP9 Expression and Secretion in Ox-LDL- and LPS-Treated HUVECs

**Figure 2:**
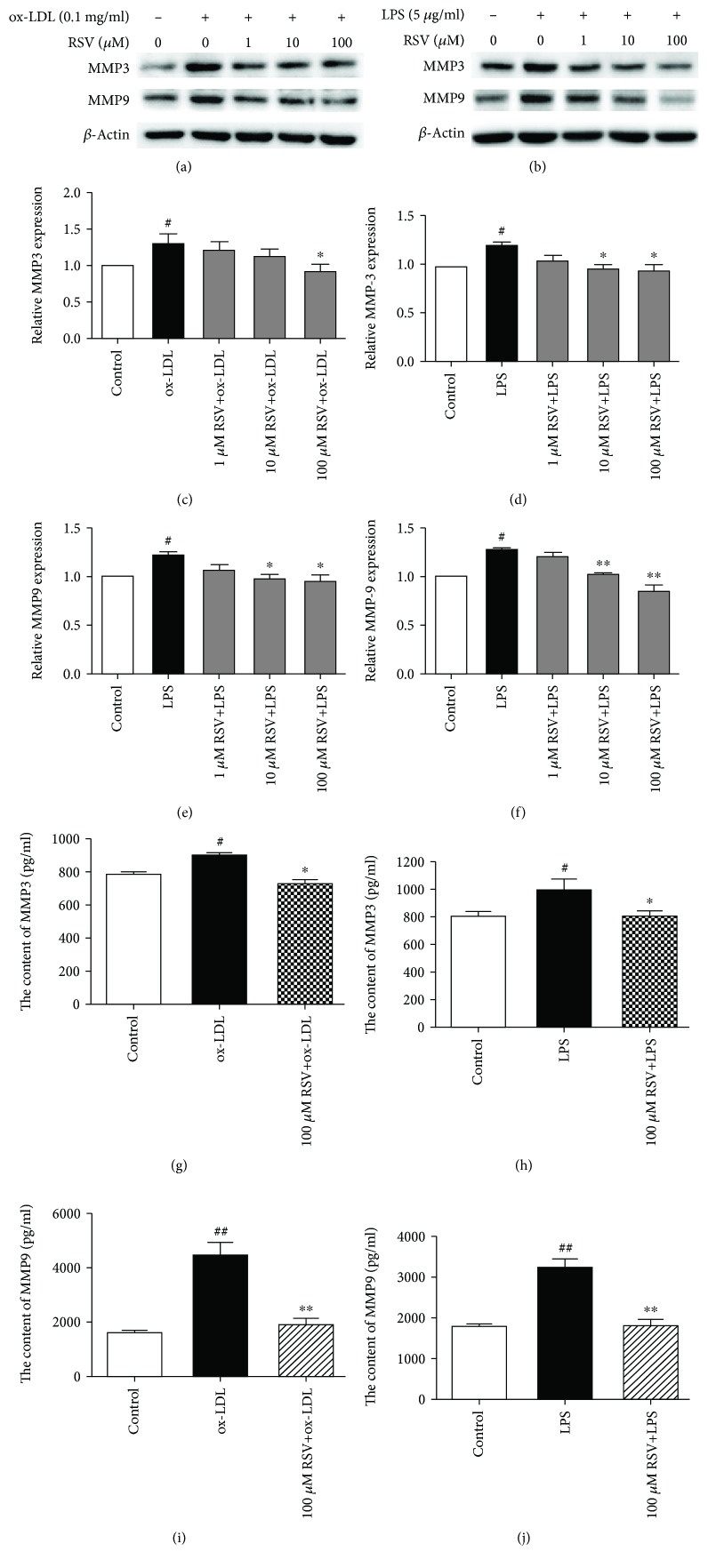
Resveratrol inhibits the expression and secretion of MMP3 and MMP9 in ox-LDL- and LPS-treated HUVECs. The cells were treated with various concentrations of resveratrol for 1 h, and ox-LDL (0.1 mg/ml) or LPS (5 *μ*g/ml) was then added for 8 h. The expression of MMP3 and MMP9 was analyzed by Western blot. The secretion of MMP3 and MMP9 was measured by an ELISA kit. The column chart represents the density analysis from three independent experiments. (a, c, e) Resveratrol suppressed MMP3 and MMP9 expression that was induced by ox-LDL. (b, d, f) Resveratrol suppressed MMP3 and MMP9 expression that was induced by LPS. (g, i) Resveratrol reduced MMP3 secretion in ox-LDL- and LPS-treated HUVECs. (h, j) Resveratrol reduced MMP9 secretion in ox-LDL- and LPS-treated HUVECs. ^#^*p* < 0.05, ^##^*p* < 0.01, significant difference between non-ox-LDL-treated cells and ox-LDL-treated cells or between non-LPS-treated cells and LPS-treated cells; ^∗^*p* < 0.05, ^∗∗^*p* < 0.01, significant difference between non-resveratrol-treated cells and resveratrol-treated cells.

Matrix metalloproteinases are key players in atherosclerosis plaque rupture, with consequent clinical cardiovascular disease. To determine whether resveratrol affects the levels of MMP3 and MMP9, we tested MMP3 and MMP9 expression in ox-LDL-treated HUVECs. As shown in Figures 2(a)–2(f), ox-LDL treatment increased MMP3 and MMP9 expression by 30.1% and 25.6%, respectively. Resveratrol (1, 10, and 100 *μ*M) dose-dependently decreased MMP3 and MMP9 expression in ox-LDL-treated HUVECs. Stimulation with LPS increased MMP3 and MMP9 expression by 21.1% and 28.9%, respectively, in HUVECs. Resveratrol (100 *μ*M) completely suppressed MMP3 and MMP9 expression in LPS-treated HUVECs.

We also evaluated MMP3 and MMP9 secretion in ox-LDL- and LPS-treated HUVECs. The content of MMP3 increased 1.15-fold in LDL-stimulated HUVECs compared with non-ox-LDL-treated cells (905.21 ± 24.77 pg/ml vs. 786.07 ± 49.28 pg/ml, respectively). Resveratrol (100 *μ*M) reduced the secretion of MMP3 in ox-LDL- and LPS-treated HUVECs. Resveratrol reduced MMP3 levels to 727.24 ± 75.98 pg/ml. We also measured MMP3 in LPS-treated HUVECs. The content of MMP3 increased 1.11-fold compared with controls (999.34 ± 134.75 pg/ml vs. 806.28 ± 64.91 pg/ml, respectively). Resveratrol decreased MMP3 levels to 806.86 ± 68.15 pg/ml, which was similar to the control group.

We then analyzed the secretion of MMP9 in ox-LDL- and LPS-treated HUVECs. The content of MMP9 increased 2.83-fold in the ox-LDL-treated group compared with the non-ox-LDL-treated group (4503.60 ± 736.77 pg/ml vs. 1587.20 ± 191.68 pg/ml, respectively). Resveratrol decreased the level of MMP9 to 1902.57 ± 364.69 pg/ml. Stimulation with LPS increased MMP9 levels 1.79-fold compared with the non-LPS-treated group (3239.96 ± 347.14 pg/ml vs. 1802.37 ± 78.49 pg/ml, respectively). Resveratrol decreased MMP9 levels to 1796.99 ± 78.49 pg/ml (Figures 2(i) and 2(j)).

### 3.3. CLI-095 Inhibited MMP3 and MMP9 Expression and Secretion in Ox-LDL- and LPS-Treated HUVECs

**Figure 3:**
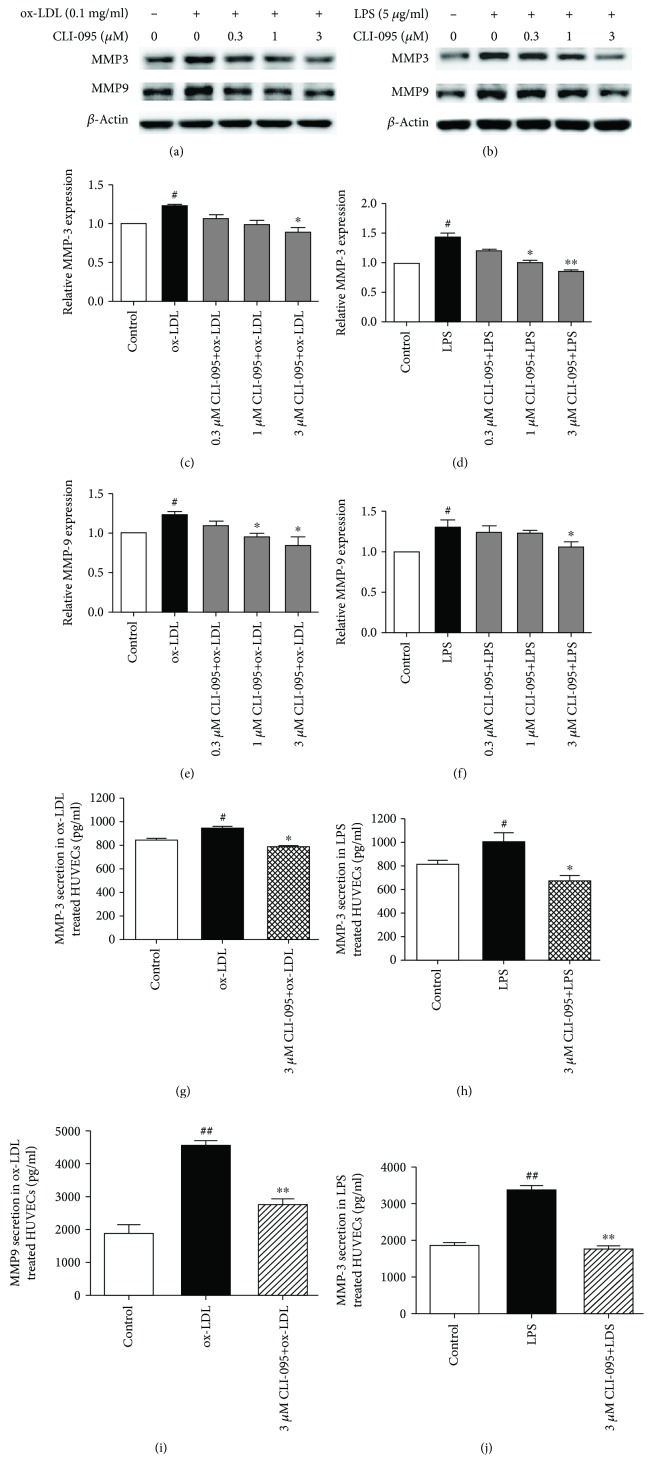
The TLR4 inhibitor CLI-095 abolishes the expression and secretion of MMP3 and MMP9 in ox-LDL- and LPS-treated HUVECs. The cells were treated with various concentrations of CLI-095 for 1 h, and ox-LDL (0.1 mg/ml) or LPS (5 *μ*g/ml) was then added for 8 h. The expression of MMP3 and MMP9 was analyzed by Western blot. The secretion of MMP3 and MMP9 was measured by an ELISA kit. The column chart represents the density analysis from three independent experiments. (a, c, e) CLI-095 suppressed MMP3 and MMP9 expression that was induced by ox-LDL. (b, d, f) Resveratrol suppressed MMP3 and MMP9 expression that was induced by LPS. (g, i) CLI-095 reduced MMP3 secretion in ox-LDL- and LPS-treated HUVECs. (h, j) CLI-095 reduced MMP9 secretion in ox-LDL- and LPS-treated HUVECs. ^#^*p* < 0.05, ^##^*p* < 0.01, significant difference between non-ox-LDL-treated cells and ox-LDL-treated cells or between non-LPS-treated cells and LPS-treated cells; ^∗^*p* < 0.05, ^∗∗^*p* < 0.01, significant difference between non-resveratrol-treated cells and resveratrol-treated cells.

To confirm that the inhibitory effect of resveratrol on MMP3 and MMP9 is related to TLR4 signaling, we used CLI-095 (a specific inhibitor of TLR4) to evaluate its inhibitory effect of MMP3 and MMP9 in ox-LDL- and LPS-treated HUVECs. As shown in Figures 3(a)–3(j), CLI-095 (0.3, 1, and 3 *μ*M) dose-dependently abolished MMP3 and MMP9 expression in ox-LDL- and LPS-treated HUVECs. We also evaluated the effect of CLI-095 on the secretion of MMP3 and MMP9. CLI-095 (3 *μ*M) decreased MMP3 levels compared with ox-LDL-treated cells (786.42 ± 24.94 pg/ml vs. 939.42 ± 38.60 pg/ml). CLI-095 (3 *μ*M) also completely inhibited MMP9 secretion that was induced by LPS. The content of MMP9 was 672.51 ± 74.29 pg/ml in CLI-095-treated HUVECs. The content of MMP9 was 1001.03 ± 132.76 in LPS-treated HUVECs.

### 3.4. Resveratrol Recovered Sirt1 Expression in Ox-LDL- and LPS-Treated HUVECs

**Figure 4:**
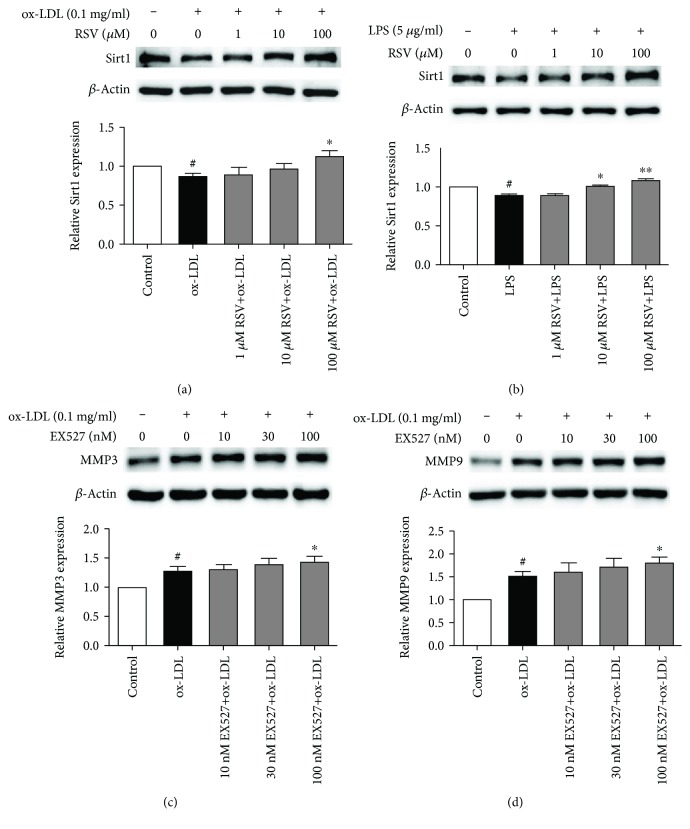
Resveratrol recovers Sirt1 expression in ox-LDL- and LPS-treated HUVECs. The cells were treated with various concentrations of resveratrol or EX527 for 1 h, and ox-LDL (0.1 mg/ml) or LPS (5 *μ*g/ml) was then added for 8 h. Sirt1 expression was analyzed by Western blot. The column chart represents the density analysis from three independent experiments. (a, b) Resveratrol recovered Sirt1 expression in ox-LDL- and LPS-treated HUVECs. (c, d) Sirt1 inhibition with EX527 increased MMP3 and MMP9 expression. ^#^*p* < 0.05, significant difference between non-ox-LDL-treated cells and ox-LDL-treated cells or between non-LPS-treated cells and LPS-treated cells; ^∗^*p* < 0.05, ^∗∗^*p* < 0.01, significant difference between non-resveratrol-treated cells and resveratrol-treated cells.

Resveratrol has been reported to be a Sirt1 agonist [26]. We investigated whether the resveratrol-induced inhibition of TLR4 signaling depends on Sirt1. As shown in Figures 4(a) and 4(b), ox-LDL and LPS treatment decreased Sirt1 expression by 13.5% and 11.3%, respectively, and resveratrol recovered Sirt1 expression in ox-LDL- and LPS-treated HUVECs. To determine whether Sirt1 influences the expression of MMP3 and MMP9 that is induced by ox-LDL, we used the Sirt1 inhibitor EX527 to suppress Sirt1 activation. As shown in Figures 4(c) and 4(d), EX527 (100 nM) significantly increased the expression of MMP3 and MMP9 that was induced by ox-LDL.

### 3.5. Resveratrol Inhibited NF-*κ*B p65 and STAT3 Phosphorylation and Nuclear Translocation in Ox-LDL- and LPS-Treated HUVECs

**Figure 5:**
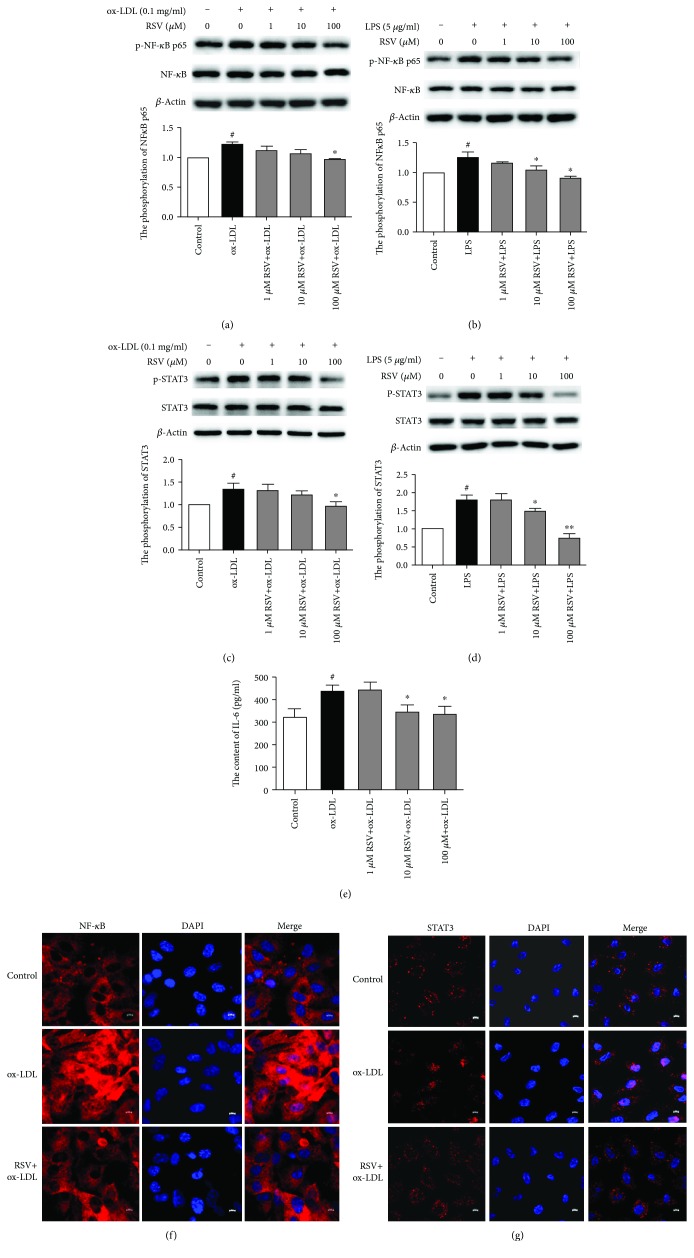
Resveratrol inhibits NF-*κ*B p65 and STAT3 phosphorylation and nuclear translocation in ox-LDL- and LPS-treated HUVECs. The cells were treated with various concentrations of resveratrol for 1 h, and ox-LDL (0.1 mg/ml) or LPS (5 *μ*g/ml) was then added for 8 h. The phosphorylation of NF-*κ*B and STAT3 was analyzed by Western blot. The column chart represents the density analysis from three independent experiments. (a, b) Resveratrol inhibited NF-*κ*B p65 phosphorylation that was induced by ox-LDL and LPS. (c, d) Resveratrol inhibited STAT3 phosphorylation that was induced by ox-LDL and LPS. (e) Resveratrol reduced IL-6 secretion that was induced by ox-LDL. (f, g) Resveratrol inhibited NF-*κ*B and STAT3 nuclear translocation that was induced by ox-LDL. ^#^*p* < 0.05, significant difference between non-ox-LDL-treated cells and ox-LDL-treated cells or between non-LPS-treated cells and LPS-treated cells; ^∗^*p* < 0.05, ^∗∗^*p* < 0.01, significant difference between non-resveratrol-treated cells and resveratrol-treated cells.

NF-*κ*B p65 is a nuclear transcription factor that is critically involved in TLR4 signaling. We explored whether resveratrol influences NF-*κ*B p65 activation in ox-LDL- and LPS-stimulated HUVECs. As shown in Figures 5(a) and 5(b), ox-LDL and LPS stimulation increased NF-*κ*B p65 phosphorylation by 21.4% and by 25.9%, respectively, in HUVECs compared with the control group, and resveratrol (100 *μ*M) completely attenuated NF-*κ*B p65 phosphorylation that was induced by ox-LDL.

STAT3 is a transcription factor that is involved in various cellular responses. We investigated whether STAT3 is activated in ox-LDL- and LPS-treated HUVECs. As shown in Figures 5(c) and 5(d), ox-LDL and LPS stimulation enhanced STAT3 phosphorylation by 33% and 79%, respectively. Resveratrol (100 *μ*M) completely abolished the phosphorylation of STAT3 that was induced by ox-LDL and LPS. In addition, we analyzed the secretion of IL-6 which is an inflammatory molecule associated with the NF-*κ*B and STAT3 pathways. As shown in Figure 5(e), the level of IL-6 increased 1.35-fold in the ox-LDL-treated group compared with the non-ox-LDL-treated group (320.58 ± 83.79 pg/ml vs. 435.93 ± 64.94 pg/ml, respectively). Resveratrol (10 and 100 *μ*M) reduced the level of IL-6, and the contents of IL-6 were 347.24 ± 64.26 pg/ml and 335.89 ± 70.43 pg/ml, respectively. There were significant differences between the resveratrol group and the ox-LDL group. Moreover, NF-*κ*B p65 and STAT3 nuclear translocation was observed by immunofluorescence. As shown in Figures 5(f) and 5(g), resveratrol inhibited NF-*κ*B p65 and STAT3 nuclear translocation that was induced by ox-LDL.

### 3.6. Effect of Resveratrol on Plasma MMP3, MMP9, and TLR4 expression in High-Fat Chow-Fed Mice

**Figure 6:**
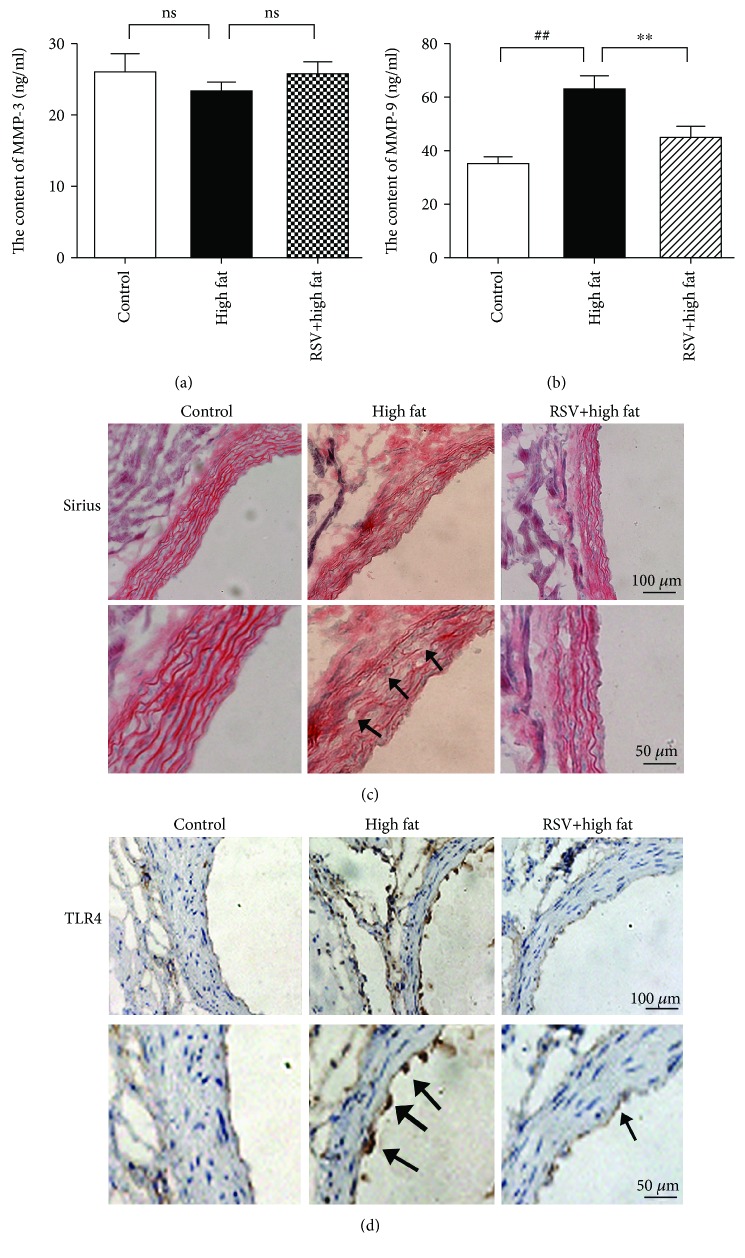
Resveratrol reduces plasma MMP3 and MMP9 levels and decreases TLR4 expression in high-fat chow-fed mice. 36-week-old male mice were sacrificed after being fed high-fat chow for 12 weeks (*n* = 12/group). Plasma MMP3 and MMP9 levels were analyzed by an ELISA kit. Sirius staining was used to detect collagen in aortic sections. Immunohistochemistry was used to analyze vascular TLR4 expression. (a) Resveratrol decreased plasma MMP9 levels but had no effect on MMP3 levels in high-fat chow-fed mice (*n* = 12). (b) Resveratrol exerted a protective effect on vascular elastic structure in high-fat chow-fed mice (*n* = 3). (c) Resveratrol reduced vascular TLR4 expression in high-fat chow-fed mice (*n* = 3). The images are representative of the results from each group.

To evaluate the vascular protective action of resveratrol *in vivo*, we performed experiments in mice. 36-week-old male mice were used in the present study. Plasma MMP3 and MMP9 levels were measured in mice that were fed normal chow, mice that were fed high-fat chow, and mice that received resveratrol and were fed high-fat chow. The content of MMP3 was 23.09 ± 4.25 ng/ml and 25.74 ± 5.93 ng/ml in the high-fat chow group and resveratrol+high-fat chow group, respectively. No difference was found between mice that were fed high-fat chow and mice that received resveratrol and were fed high-fat chow (Figure 6(a)). MMP9 secretion significantly increased in high-fat chow-fed mice. The content of MMP9 was 63.06 ± 16.12 ng/ml in the high-fat chow group and 45.15 ± 13.09 ng/ml in the resveratrol+high-fat chow group. A significant difference was found between the high-fat chow group and resveratrol+high-fat chow group (Figure 6(b)). The content of MMP9 was 35.44 ± 7.1 ng/ml in the normal chow group.

Next, we analyzed the effect of resveratrol on vascular structure in high-fat chow-fed mice. Sirius staining was used to observe changes in collagen in the aorta of mice. As shown in Figure 6(c), the arrangement of collagen was disordered and fractured in high-fat chow-fed mice compared with the normal chow group. The arrangement of collagen was regular and less fractured in resveratrol-treated mice. We also evaluated TLR4 expression in aortic sections by immunohistochemistry. The level of TLR4 expression was significantly higher in the aorta in high-fat chow-fed mice compared with the normal chow group and resveratrol-treated group (Figure 6(d)). This suggests that resveratrol had a protective effect against high-fat diet-induced vascular damage.

## 4. Discussion

Atherosclerosis is a chronic inflammatory disease that is characterized by lipid accumulation in vessels and inflammation. Recent studies have demonstrated a critical role for TLR4 in the initiation and progression of inflammation in atherosclerosis [27, 28]. Ox-LDL is an important risk factor for the development and progression of atherosclerosis. In the present study, we explored the effects of resveratrol on the TLR4-mediated expression and secretion of metalloproteases and the underlying mechanism. Matrix metalloproteinases are a family of extracellular zinc proteases that process protein components of the ECM. The various mediators of inflammation, such as TNF*α* and IL-1*β*, can stimulate MMP expression. The gene expression of MMPs is related to the NF-*κ*B pathway. The activity of MMPs promotes vascular remodeling by degrading ECM composition [29]. Increases in MMPs facilitate endothelial dysfunction, inflammation, and thrombosis in atherosclerosis. We found that both resveratrol and the TLR4-specific inhibitor CLI-095 inhibited the expression and secretion of MMP3 and MMP9 that were induced by ox-LDL and LPS. This suggests that increases in MMP3 and MMP9 are associated with TLR4 activation, and resveratrol reverses this effect by inhibiting TLR4 signaling. A recent study reported that TLR2 and TLR4 deficiency markedly reduced atherosclerosis that was induced by oral bacteria [30]. The effect of ox-LDL on human atherosclerosis may be at least partially mediated by the TLR4 pathway [31, 32]. The present study provides further evidence that resveratrol inhibits TLR4 activation and reduces the expression and secretion of MMP3 and MMP9 in ox-LDL-treated HUVECs. These results are consistent with previous studies. Moreover, we also found that the secretion of MMP9 is much higher than that of MMP3 at the cellular level and in plasma in mice. This suggests that MMP9 might play a more important role than MMP3 in the vascular inflammatory response *in vivo*. A previous study that followed patients for 10 years found that high MMP9 levels were associated with a higher incidence of stroke and cardiovascular death [33].

In the present study, we found that both ox-LDL- and LPS-induced NF-*κ*B and STAT3 activation were associated with TLR4 signaling, and resveratrol attenuated the phosphorylation of both NF-*κ*B and STAT3. The inhibition of TLR4 with CLI-095 abolished NF-*κ*B and STAT3 phosphorylation. This strongly suggests that both NF-*κ*B and STAT3 are involved in the TLR4-mediated inflammatory response in ox-LDL-treated HUVECs. These results are consistent with our previous study, in which both NF-*κ*B and STAT3 were linked to TLR4 activation in high glucose-treated HUVECs [14]. Recently, we reported that resveratrol inhibiting TLR4 expression was associated with suppressing Akt phosphorylation in ox-LDL-stimulated platelets [34]. Akt is an upstream molecule on the NF-*κ*B pathway. Akt phosphorylates IKK*α* and subsequently phosphorylates NF-*κ*B p65 [35].

The NF-*κ*B family of proteins is involved in regulating innate and adaptive immune responses and cell survival. NF-*κ*B and inflammation constitute a positive feedback loop [36]. NF-*κ*B is activated in response to stress, obesity, infectious agents, and environmental stimuli that commonly contribute to chronic inflammation [37, 38]. Moreover, STAT3 is another important transcription factor that plays a critical role in inflammation-related diseases that involve cytokine signal transduction, oxidative stress, and apoptosis [39]. Our results indicate that resveratrol can protect against ox-LDL- and LPS-induced inflammation by suppressing the NF-*κ*B and STAT3 pathway in HUVECs.

Sirt1 is a target molecule of resveratrol [40, 41]. The present results provide further evidence that both ox-LDL and LPS stimulation reduce Sirt1 expression, and resveratrol recovers Sirt1 expression. In contrast, the inhibition of Sirt1 activation with EX527 enhanced ox-LDL-induced MMP3 and MMP9 expression. This suggests that the resveratrol-induced inhibition of TLR4 at least partially depends on Sirt1 activation. We recently reported that the combination of the Sirt1 inhibitor EX527 and resveratrol impeded the inhibitory effect of resveratrol on TLR4 expression in ox-LDL-activated platelets [34].

The limit of this study is that we use a higher concentration of resveratrol, which may have a negative impact on clinical applications. Previous studies showed that the experimental concentration range of resveratrol is very broad. The concentration of resveratrol is 32 nM-100 *μ*M *in vitro* and 100 ng-1500 mg/kg (body weight) *in vivo* [42]. Animal studies have shown that no side effect was found after 300 mg/kg (body weight) of resveratrol per day for 4 weeks in rats [43]. However, the safety and efficacy of resveratrol remain to be further evaluated.

**Figure 7:**
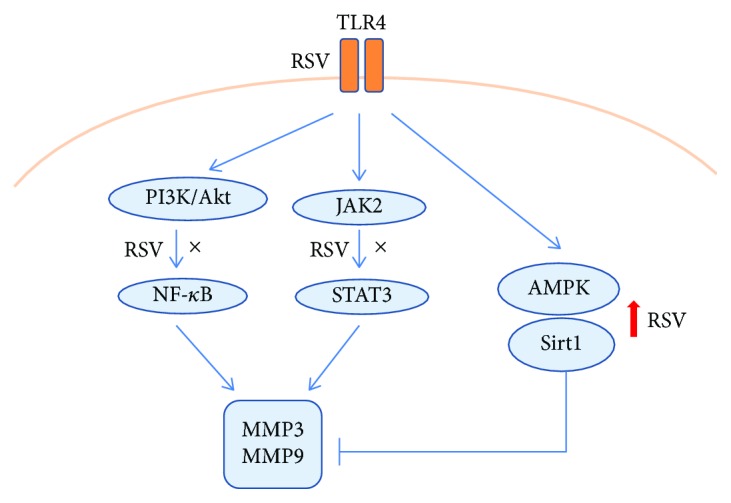
The summary of the molecular effects of resveratrol. Resveratrol inhibits MMP3 and MMP9 expression and secretion by suppressing TLR4/NF-*κ*B/STAT3 activation and increases Sirt1 expression in ox-LDL-treated HUVECs.

In conclusion, the present study showed that resveratrol inhibited MMP3 and MMP9 expression and secretion in ox-LDL- and LPS-treated HUVECs. The mechanism of action of resveratrol was associated with the suppression of TLR4-mediated NF-*κ*B p65/STAT3 activation and the recovery of Sirt1 expression in ox-LDL- and LPS-treated HUVECs. Resveratrol decreased TLR4 expression and MMP9 secretion in high-fat chow-fed mice and conferred protection against vascular inflammation in mice. We summarized the above studies as shown in Figure 7.

## Data Availability

The data used to support the findings of this study are available from the corresponding author upon request.
